# Retinal expression of small non-coding RNAs in a murine model of proliferative retinopathy

**DOI:** 10.1038/srep33947

**Published:** 2016-09-22

**Authors:** Chi-Hsiu Liu, Zhongxiao Wang, Ye Sun, John Paul SanGiovanni, Jing Chen

**Affiliations:** 1 Department of Ophthalmology, Boston Children’s Hospital, Harvard Medical School, Boston, Massachusetts 02115, USA; 2 Section on Nutritional Neuroscience, National Institute on Alcohol Abuse and Alcoholism, Bethesda, MD 20892, USA

## Abstract

Ocular neovascularization is a leading cause of blindness in proliferative retinopathy. Small non-coding RNAs (sncRNAs) play critical roles in both vascular and neuronal development of the retina through post-transcriptional regulation of target gene expression. To identify the function and therapeutic potential of sncRNAs in retinopathy, we assessed the expression profile of retinal sncRNAs in a mouse model of oxygen-induced retinopathy (OIR) with pathologic proliferation of neovessels. Approximately 2% of all analyzed sncRNAs were significantly altered in OIR retinas compared with normoxic controls. Twenty three microRNAs with substantial up- or down-regulation were identified, including *miR-351*, *-762*, *-210*, *145*, *-155*, *-129-5p*, *-150*, *-203*, and *-375*, which were further analyzed for their potential target genes in angiogenic, hypoxic, and immune response-related pathways. In addition, nineteen small nucleolar RNAs also revealed differential expression in OIR retinas compared with control retinas. A decrease of overall microRNA expression in OIR retinas was consistent with reduced microRNA processing enzyme Dicer, and increased expression of *Alu* element in OIR. Together, our findings elucidated a group of differentially expressed sncRNAs in a murine model of proliferative retinopathy. These sncRNAs may exert critical post-transcriptional regulatory roles in regulating pathological neovascularization in eye diseases.

Proliferative retinopathy is a common cause of vision loss and characterized by pathologic retinal neovascularization extending into the vitreous body in the eye. Both diabetic retinopathy (DR) and retinopathy of prematurity (ROP) display cardinal features of proliferative neovascularization of the inner retinal vascular bed^1^,^2^,^3^. Development of pathologic retinal neovascularization is linked with secretion of hypoxia-induced pro-angiogenic factors such as vascular endothelial growth factor (VEGF) and erythropoietin. These factors promote consequent formation of aberrant neovessels, eventually leading to retinal hemorrhage, detachment and neurodegeneration^4^,^5^. Current surgical interventions (e.g., vitrectomy) and laser treatments (including photocoagulation) for proliferative retinopathy are invasive and of limited efficacy. For instance, laser photocoagulation, the standard treatment for proliferative retinopathy by ablating peripheral retinas and neovessels, can improve retinal hypoxic condition, yet is often associated with permanent peripheral vision loss^3^,^6^,^7^. Anti-VEGF therapy is effective in treating pathologic neovascularization in both ROP and DR^7^,^8^. However, its long term safety on vessel and neuronal health and potential systemic effects are still being evaluated, as VEGF is essential for normal vessel and neuronal survival^7^,^9^. Therefore, it is critical to identify additional controlling factors that may impact ocular neovascularization in order to develop new therapies.

Vascular growth is highly dependent on oxygen demand and concentration in the microenvironment, thus retinal hypoxia and resulting ischemia play significant roles in retinopathy development^2^,^3^. Pathologic retinal neovascularization can be modeled in mice with an oxygen-induced proliferative retinopathy (OIR) model^10^. This model mimics the proliferative stage of ROP and DR^10^, and is widely used to study retinopathy pathogenesis and test potential anti-angiogenic therapies, including the early experimental work evaluating anti-VEGF therapies^11^. This model is also beneficial for identifying additional pathogenic factors that may regulate retinal neovascularization, including post-translational RNA control beyond regulation by proteins.

Small non-coding RNAs (sncRNAs), such as microRNAs (miRNAs) and small nucleolar RNAs (snoRNAs), have critical biological functions as regulatory elements influencing human development and diseases. MiRNAs are the most extensively studied sncRNAs; these are highly conserved molecules of ∼21–25 nucleotides that function mainly in mRNA silencing capacity. Post-transcriptional regulation of gene expression by miRNAs plays a key role in ocular tissue development and in angiogenesis^12^,^13^, suggesting a potential role of miRNAs in proliferative retinal neovascularization. SnoRNAs are another class of newly discovered yet less studied sncRNAs varying from 60 to 300 nucleotides in length. SnoRNAs play important roles in post-transcriptional RNA modification and may impact oncogenesis as well as pathogenesis of some hereditary and autoimmune diseases, such as Prader-Willi syndrome and multiple sclerosis^14^,^15^, yet their roles in retinopathy is not clear.

In this study, we used a mouse model of OIR to identify sncRNAs responsive to perturbations associated with pathologic retinal angiogenesis. Analysis in the mouse model is important as there are few human sncRNA retinal expression data available from patients with proliferative retinopathy. SncRNAs significantly regulated in this murine model may be potential mediators of hypoxia-driven regulation of retinal neovascularization. Our data revealed twenty three miRNAs and nineteen snoRNAs with significantly altered expression levels in OIR mouse retinas as compared with those from normoxic control mice. Pathway analyses identified hypoxia and angiogenesis related genes as putative miRNA targets in OIR retinas, shown in a graphic network of miRNA-gene interaction. In addition, our analysis in the transcriptomic profiles characterizing pathologic angiogenesis in the human vascular retina and choriocapillaris also strengthened inferences on the influence of miRNAs as key regulators in human vascular eye diseases. Moreover, we observed decreased overall miRNA expression in OIR retinas consistent with reduced miRNA processing enzyme Dicer, and increased expression of *Alu* element. Overall, this study provides a detailed analysis of sncRNA expression in a murine model of proliferative retinopathy. Further investigation of these candidates may help elucidate the post-transcriptional regulatory events leading to pathologic neovascularization in proliferative retinopathy.

## Results

### Microarray analysis of miRNA expression profile in mouse OIR model

**Figure 1:**
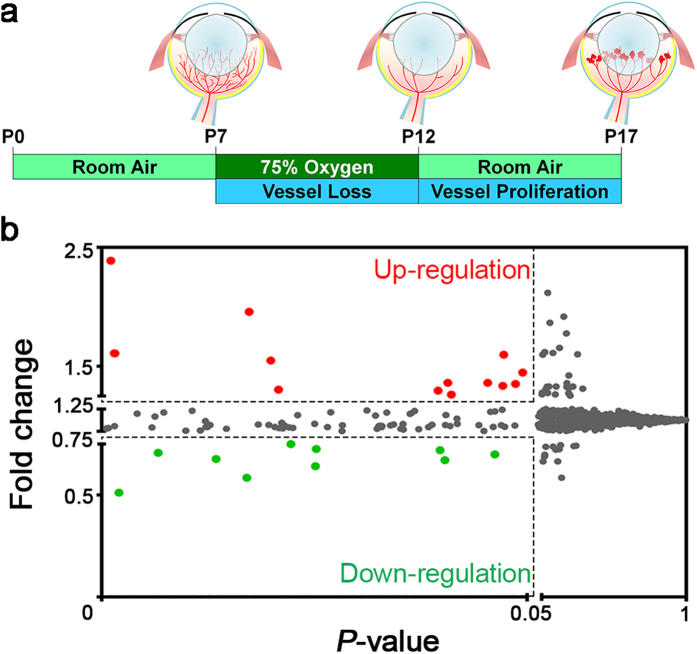
MiRNA levels were altered in the mouse retinas with oxygen-induced retinopathy (OIR). (**a**) Schematic diagram of the OIR model. Neonatal mice were exposed to 75% oxygen from postnatal day (P) 7 to P12 and returned to room air from P12 to P17 to induce maximum pathologic neovascularization at P17. Total RNAs were isolated from OIR-exposed or age-matched normoxic control mouse retinas at P17 (n = 3 per group) and subsequently applied to Affymetrix GeneChip miRNA array chips for analyzing small non-coding RNAs (sncRNAs), including mature miRNAs, pre-miRNAs (stem-loops), and snoRNAs. There were 1,412 mouse (*Mus musculus*) miRNAs on the array chip. (**b**) A dot plot illustrates the mean fold changes of miRNA expression in OIR compared with normoxic retinas versus their *P*-value. Each dot represents one miRNA (including pre-miRNA). Among all analyzed miRNAs, 0.92% of them (13 miRNAs, as red dots) were up-regulated at more than 1.25-fold, and 0.71% (10 miRNAs, as green dots) were down-regulated in OIR retinas at less than 0.75-fold.

The mouse OIR model exposes mouse pups to 75% oxygen from postnatal day (P) 7 to 12. After returning to room air at P12, the relative hypoxia stimulates retinal ischemia and pathologic neovascularization. Maximal pathologic retinal angiogenesis can be observed at P17, mimicking pathologic retinal vessels as seen in ROP and DR (Fig. 1a)^10^. To identify the difference in miRNA expression profiles, we performed microarray analysis of miRNAs using total RNA isolated from P17 mouse OIR retinas and age-matched normoxic controls. The array chip contains probes for 1,412 mouse mature and precursor (stem-loop) miRNAs, all of which were included in the significance analysis. MiRNAs with significantly altered levels (*P* < 0.05) in OIR retinas compared with controls were identified. Among all miRNAs analyzed, approximately 1% of them (13 miRNAs) were up-regulated in OIR retinas with fold change greater than 1.25-fold, and 0.71% of them (10 miRNAs) were down-regulated at less than 0.75-fold (Fig. 1b).

### Expression of miRNAs was altered in mouse OIR retinas

**Figure 2:**
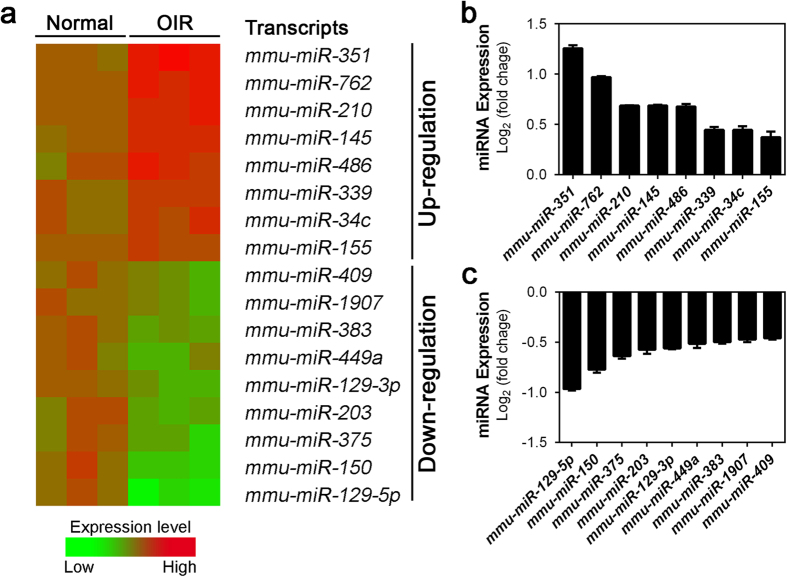
Expression levels of significantly altered miRNAs in OIR retinas. (**a**) A heat map illustrates selected mature miRNAs with levels significantly changed in OIR retinas at P17, as analyzed from array results. Red indicates up-regulated expression levels; green indicates down-regulated expression levels, as compared with mean levels from three normoxic control samples. (**b,c**) Significant fold-changes of miRNAs in OIR retinas versus normoxic controls expressed in log_2_ scale. Eight miRNAs (*miR-351*, *-762*, *-210*, *-145*, *-486*, *-339*, *-34c*, and *-155*) were substantially up-regulated (**b**), and nine miRNAs (*miR-129-5p*, *-150*, *-375*, *-203*, *-239-3p*, *-449a*, *-383*, *-1907* and *-409*) were substantially down-regulated in OIR retinas (**c**). Data were presented as mean ± standard error of the mean (SEM).

Among the thirteen miRNAs that showed significantly up-regulated in P17 OIR retinas, eight of them (*mmu-miR-351*, *-762*, *-210*, *-145*, *-486*, *-339*, *-34c* and *-155*) were mature miRNAs as shown in the heat map (Fig. 2a) with their specific expression fold changes shown in a bar graph (Fig. 2b). Among these, *mmu-miR-351* displayed the greatest increase of approximately 2.4-fold (log_2_ ratio was ∼1.25) (Fig. 2a,b and Table 1). The second most significantly up-regulated miRNA was *mmu-miR-762* with nearly 2-fold change (Fig. 2a,b and Table 1). Three more miRNAs (*mmu-miR-210*, *-145*, and *-486*) also revealed substantial up-regulation in the OIR retinas with ∼1.5-fold change (representing log_2_ ratio >0.58) in the OIR model (Fig. 2a,b and Table 1).

Moreover, we found that ten miRNAs were down-regulated while nine of these (*mmu-miR-129-5p*, *-150*, *-375*, *-203*, *129-3p*, *-449a*, *-383*, *-1907* and *-409*) were mature miRNAs shown in the heat map and bar graph (Fig. 2a,c). Among these, *mmu-miR-129-5p* and *-150* were the two most substantially decreased miRNAs, showing more than 40% of down-regulation (log_2_ ratio < −0.7, representing <0.6-fold change) compared with the age-matched normoxic retinas (Fig. 2a,c and Table 1). In addition four more miRNAs (*mmu-miR-375*, *-203*, *-129-3p* and *-449a*) showed significant down-regulation in mouse OIR retinas at log ratio <−0.5 (Fig. 2a,c and Table 1).

### Expression profile of snoRNAs was changed in OIR retinas

**Figure 3:**
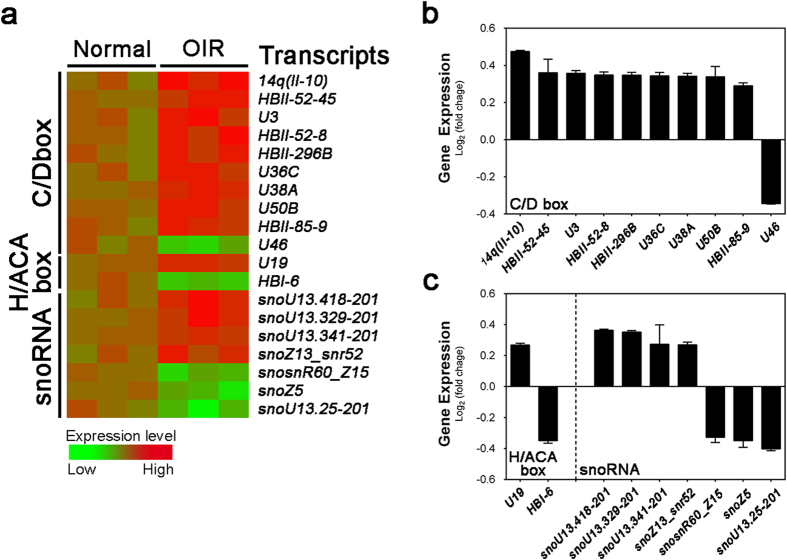
OIR-induced alteration of small nucleolar RNAs (snoRNAs) in mouse retinas. (**a**) A heat map shows snoRNAs (C/D box, H/ACA box and unclassified snoRNAs) labeled as C/D box, H/ACA box and snoRNA respectively, with significantly changed levels (*P* < 0.05) in OIR retinas compared with age-matched normoxic controls from miRNA array data. Red color indicates up-regulated expression levels in OIR; green color indicates down-regulated expression levels. (**b,c**) Significant fold-changes of snoRNAs in OIR retinas versus normoxic controls expressed in log_2_ scale. Thirteen snoRNAs (9 C/D box snoRNAs, 1 H/ACA box snoRNA, and 4 unclassified snoRNAs) were substantially up-regulated (**b**), and five snoRNAs (1 C/D box snoRNA, 1 H/ACA box snoRNA, and 3 unclassified snoRNAs) were substantially down-regulated in OIR retinas (**c**). Data were presented as mean ± SEM.

In addition to miRNAs, we also analyzed in OIR retinas the differential expression profiles of the snoRNAs, another class of small non-coding RNAs associated with nucleotide chemical modification, such as methylation and pseudouridylation, by guiding and tethering partner enzymes to specific sites on RNA targets^16^,^17^,^18^. The array chip has 2,302 probes for snoRNA and 19 probes in the array revealed significant altered expression in P17 OIR retinas compared with normoxic controls (Fig. 3a and Table 2). Fourteen of them were up-regulated and five were down-regulated in OIR retinas. Based on the nucleotide sequence characteristics, ten of these altered snoRNAs were in the C/D box family (Fig. 3b), with two conserved motifs, C (consensus RUGAUGA) and D (CUGA), at the 5′ and 3′ ends respectively. Two snoRNAs were recognized as H/ACA box snoRNAs with conserved motifs H box (ANANNA) and the ACA box (ACA). C/D box and H/ACA box snoRNAs guide 2′-O-methylation and pseudouridylation, respectively^16^,^17^. The rest seven were unclassified snoRNAs (Fig. 3c). Among the 19 snoRNAs, seven of them have reported target RNAs, whereas twelve snoRNAs have no known targets and hence are orphan snoRNAs (Table 2). Although these snoRNA probes are of human sequence, mouse orthologs of them largely exist and may harbor unknown function to be explored.

We found that the most up-regulated snoRNA is *14q(II-10)*, at ∼1.4-fold change (log_2_ ratio was ∼0.5) in OIR retinas compared with normoxic controls (Fig. 3a,b and Table 2). *14q(II-10)* (*SNORD114-10*) is a C/D box snoRNA, whose human form is located on chromosome 14q32. The mouse *SNORD114-10* ortholog gene, *Gm25224*, shares 68% of identity and 100% of coverage with the human form, and is encoded in homologous loci on mouse chromosome 12. Another C/D box snoRNA, *HBII-296B* (*SNORD91B*), revealed up to 1.27-fold change and 90% of identity to its mouse ortholog *Gm22771*. In addition, *U19* (*SNORA74*), the most substantially altered H/ACA snoRNA in the array, showed 20% increase in OIR retinas compared to normoxic retinas; moreover, its mouse ortholog, *Gm23946*, shares 78% of identity and 97% of coverage. Our findings indicated that these altered snoRNAs may exert post-translational co-regulatory roles in mediating OIR response, potentially through altering ribosome biogenesis pathways^16^,^17^.

### MiRNA biogenesis was decreased in OIR retinas

**Figure 4:**
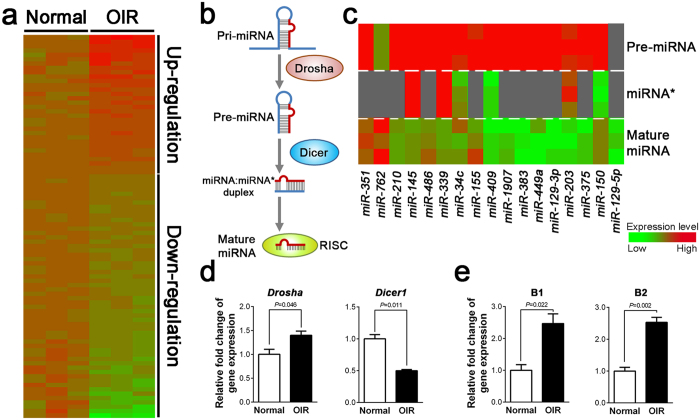
Altered expression levels of RNA processing enzymes *Drosha* and *Dicer*, and *Alu*-like RNAs in OIR are consistent with relatively down-regulated mature miRNA levels compared with pre-miRNAs. (**a**) Heat map illustration of miRNA array data shows those miRNAs with significantly changed levels (*P* < 0.05) in OIR mouse retinas compared with age-matched normoxic retinas. Red color indicates up-regulated expression levels in OIR; green color indicates down-regulated expressing levels. (**b**) Schematic diagram of the miRNA biogenesis. MiRNA genes are usually transcribed by RNA polymerase II to the primary miRNAs (pri-miRNAs), which are processed by Drosha, yielding precursor miRNAs (pre-miRNAs) in the nucleus. After being exported to the cytoplasm, the pre-miRNAs are cleaved by Dicer to generate sense (mature) miRNA:anti-sense miRNA* duplexes. Following unwinding of the miRNA duplexes, the single-strain mature miRNAs are assembled into the RNA-induced silencing complex (RISC) for binding to their target mRNAs to mediate transcription repression or degradation. (**c**) Heat map shows relatively up-regulated pre-miRNA levels in P17 OIR mouse retinas compared with normoxic control retinas, along with corresponding antisense (*) and sense (mature) miRNA levels. Red indicates up-regulated expression level; green indicates down-regulated expression level. (**d**) Gene expression levels of *Drosha* and *Dicer1* in normoxic (Normal) and OIR mouse retinas were measured by RT-qPCR. (**e**) Expression levels of transcription repressive *B1*/*B2* in normal and OIR retinas. Results were normalized to housekeeping gene *18S* and to expression levels in age-matched normoxic controls. Data were presented as mean ± SEM, n = 6 per experimental group.

From our analysis of OIR miRNA expression profile, we observed global miRNA down-regulation in OIR retinas (Fig. 4a). For all mouse miRNA probes, including mature and precursor miRNAs (pre-miRNAs), more than half (∼56%) of probes showed decreased expression levels in OIR retinas compared with the average fold change of normoxic controls. Among all significantly changed mature miRNAs, 34.6% were up-regulated and 65.4% were down-regulated, suggesting that miRNA processing pathway might be affected in OIR.

To determine whether the miRNA biogenesis process (Fig. 4b) is affected in OIR, we next analyzed the expression of pre-miRNAs and mature-miRNAs from the significantly altered miRNAs (Table 1). The results showed remarkable down-regulation of mature RNAs, compared with their respective pre-miRNAs in OIR (Fig. 4c). The decrease of overall miRNA expression and particularly mature miRNAs might be caused by altered levels of key RNA processing enzymes, such as Drosha and Dicer (Fig. 4b), both ribonuclease III responsible for processing primary miRNAs (pri-miRNAs) and cleaving pre-miRNAs respectively. We thereby examined the expression of *Drosha* and *Dicer1* mRNAs in OIR and normoxic retinas using q-PCR. *Drosha* was slightly increased in OIR retinas; whereas expression of *Dicer1* was significant decreased (∼50%) in the OIR retinas (Fig. 4d), indicating that insufficient Dicer expression may explain in part the decreased mature miRNA expression levels.

Previous studies in human age-related macular degeneration indicated that DICER deficiency caused accumulation of *Alu* RNA, leading to retinal cell death^19^. *Alu* elements, derived from the small cytoplasmic 7SL RNA, are also post-transcriptional regulators of gene expression through alternative splicing and RNA editing^20^. Since we observed the decrease of *Dicer1* in OIR, we next evaluated whether the expression levels of mouse *Alu*-equivalent B1 and B2 elements were changed in OIR. We found that both B1 and B2 RNAs were significantly increased in OIR retinas (Fig. 4e), suggesting the possibility of coordinated regulation of *Alu* RNA and their potential roles in proliferative retinopathy.

### *In Silico* miRNA-to-target networks in regulating pathologic neovascularization in OIR

**Figure 5:**
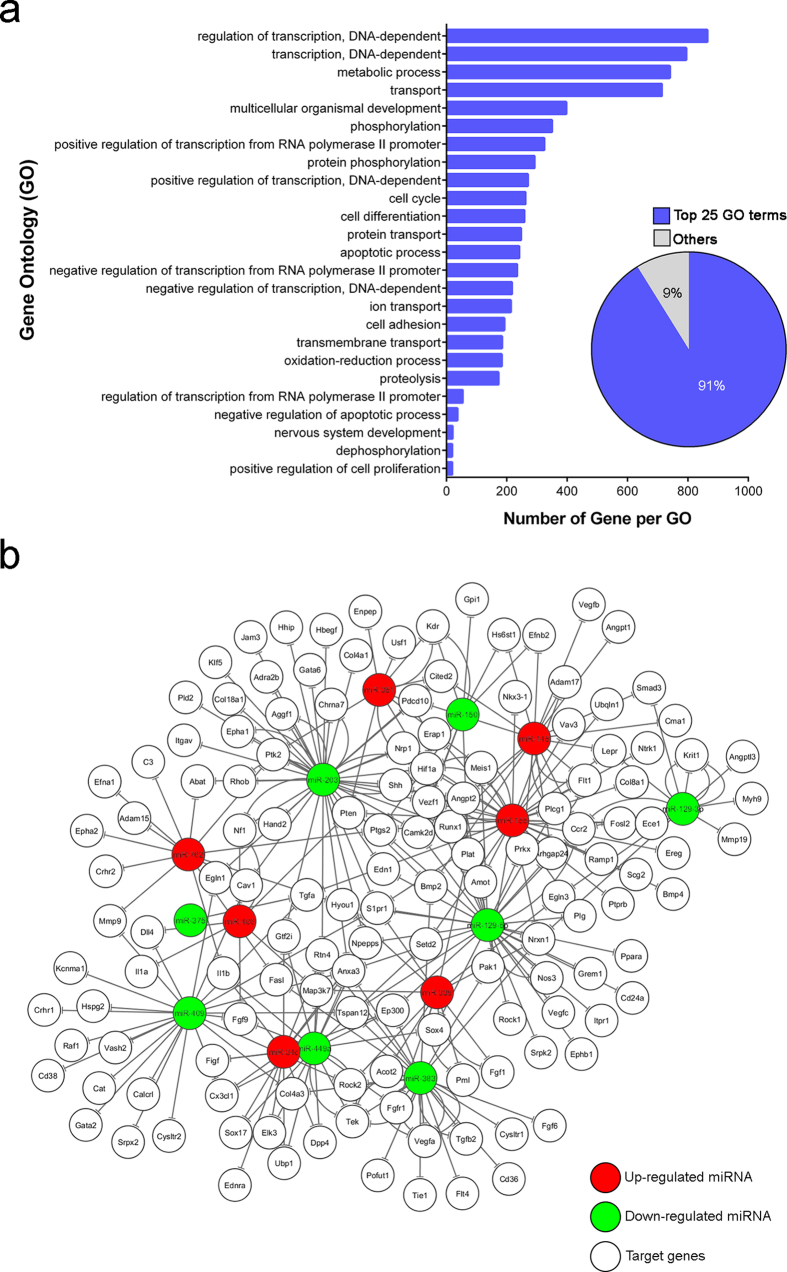
Gene ontology (GO) category analysis of miRNA targeted genes. The significantly altered miRNAs in OIR were subjected to target prediction by TargetScan and miRanad-mirSVR, with GO annotation and singular functional enrichment analysis using GeneCodis3. (**a**) Top 25 GO terms included 91% of the putative target genes. The vertical axis represents the GO categories; the horizontal axis indicates the number of genes per GO. (**b**) Summary of miRNA-target gene network shows the miRNAs and their potential target genes which may response to hypoxia and direct angiogenesis in OIR. Up- (red) or down-regulated (green) miRNAs in OIR were subjected to GO annotation and singular functional enrichment analysis using TargetScan, miRanad-mirSVR and GeneCodis3 and plotted with their potential targets using Cytoscape (version 3.2.1). Angiogenesis-related genes, such as those relevant to VEGF (*Vegfa*, *Flt1*, and *Kdr*) and ANGPT-TIE (*Angpt1*, *Angpt2* and *Tek*) signaling, and genes in hypoxia response (*Hif1a* and *Ep300*), may be regulated by miRNAs altered in OIR to impact pathologic retinal neovascularization.

Having identified multiple miRNA alteration in OIR, we next evaluated their potential mRNA targets that may impact neovascularization. Specifically we composed a molecular network spanning direct miRNA-mRNA targets in OIR by using bioinformatics databases TargetScan and miRanada-mirSVR, following by singular enrichment analysis performed by GeneCodis3 program^21^,^22^,^23^. A total of 7,998 genes, which might be targeted by those miRNAs with significant differential expression in OIR (identified in Table 1), were found with gene ontology (GO) annotation in biological process categories. Over 90% (7,289) of the putative target genes were classified in the top 25 GO categories (Fig. 5a). Most of the targets were shown related to transcription regulation, cellular component transport, cell cycle and development. Moreover, the enrichment analysis showed that 137 target genes were annotated in angiogenic- and hypoxic-related GO categories. Among them, 105 genes were related to angiogenesis, including both positive and negative regulation of angiogenesis; and 41 genes were annotated with response to hypoxia.

After combining the prediction of miRNA-gene interaction with the functional GO analysis for the differentially expressed miRNAs in a graphic form, we obtained a visual illustration of the miRNA-target gene network associated with angiogenesis and hypoxia in the OIR (Fig. 5b). This integration illustrated a complex cross-interacting network of identified miRNAs and their predicted targets which may participate in angiogenesis and be involved in pathogenesis of proliferative retinopathy. Some of the putative target genes may be regulated by several miRNAs, and involved in multiple GO categories.

### Identification of OIR-responsive miRNA targets in transcriptome profile of human neovascular eye diseases

Our findings accentuated the potential regulatory roles of miRNAs in retinopathy disease progression, suggesting that miRNAs would be critical regulators of transcriptomic profiles and biomarkers linked to the human vascular eye diseases. In order to identify such promising miRNA-based biomarkers, we used information on the targets of the OIR-responsive miRNAs (Table 1) supported by strong experimental evidence in miRTarBase 6.1 to filter disease-defining constituents of transcriptomic profiles from human retinal specimens containing: 1) active and quiescent fibrovascular membranes (FVMs) of people with proliferative diabetic retinopathy (PDR)^24^; and, 2) choroidal neovascular (CNV) membranes of people with neovascular age-related macular degeneration (AMD)^25^ (Table 3). Fourteen of the OIR-responsive miRNAs yielded findings in the analyses. Eight of them showed validated gene targets differentially expressed in retinas of people with PDR, whereas six OIR-responsive miRNAs had targets in active FVMs. In patient samples with neovascular AMD, six of the OIR-responsive miRNAs had experimentally validated targets in genes differentially expressed in neural retinas, and six of the altered miRNAs in OIR showed gene targets in RPE/choroid complexes. After probing potential targets of OIR-responsive miRNAs in the context of comparative neovascular AMD and PDR transcriptome analyses, we investigated functional enrichment of the 38 pertinent targets identified both as validated OIR-responsive miRNA targets and dysregulated molecules in PDR and neovascular AMD by using GeneCodis3. The strongest enrichment from the GO biological processes database existed for transcriptional regulation, signal transduction, development, and blood coagulation, with 7 of the 38 pertinent constructs defining these processes (Table S1). Focal adhesion and p53 signaling showed strong enrichment in human KEGG pathways from the GeneCodis3 database, with 5 targets of OIR-responsive miRNAs differentially expressed in human retinal specimens with PDR, and 4 targets for neovascular AMD (Table S2). Together our analysis indicated that miRNAs may define gene expression signature in human retinal tissues manifesting microvascular pathophysiology, offering promise for development of global biomarkers and/or prognostic indicators.

## Discussion

MiRNAs play crucial roles in the biological development and cellular function, such as neurogenesis, angiogenesis, inflammation, and metabolism^26^,^27^,^28^,^29^. Dysregulation of miRNA impacts many diseases, including cancers, neurological and cardiovascular diseases; thereby miRNAs are being developed as important biomarkers for these diseases^28^,^30^,^31^. In this study, we demonstrated differential expression profiles of miRNA in an oxygen-induced proliferative retinopathy model by microarray analyses (Fig. 1). Our data identified numerous miRNAs that were significantly altered in mouse retinas with induced proliferative retinopathy (Table 1 and Fig. 2), suggesting that these miRNAs may be potential regulators of abnormal pathologic vascular formation in proliferative retinopathy. Furthermore, our data revealed a global miRNA down-regulation in the mouse OIR retinas likely caused by decreased expression of *Dicer1* (Fig. 4a–d). Previous studies showed that retinal Dicer-knockout mice exhibit global miRNA down-regulation which compromises the normal retinal development^32^,^33^,^34^, indicating that our observation of *Dicer* deficiency may partially explain impaired vascular and neuronal development in OIR retinas. Consistent with our findings, trends of decreased overall retinal miRNA expression can be derived from previous works utilizing different rodent models of oxygen-induced ischemic retinopathy associated with retinal hypoxia^35^,^36^. Hypoxia is a key regulator in altering miRNA expression profiles in human cancers via decreasing miRNA biogenesis and suppression of *Dicer* through an epigenetic regulation mechanism^37^. Together these findings suggest that miRNA biogenesis is substantially dysregulated in retinopathy, likely modified by retinal hypoxia and resultant *Dicer* suppression.

In addition to processing miRNAs, DICER1 also processes degradation of *Alu* RNA element. *Alu* RNA accumulation and DICER1 deficit stimulates retinal pigmented epithelium degeneration and geography atrophy in age-related macular degeneration^19^. In diabetic patients and murine diabetic models, *Dicer* exhibited reduced expression and *Alu* RNA was up-regulated^38^, implicating that disrupted miRNA biogenesis and *Alu* toxicity may be associated with the development of diabetic retinopathy. Apart from the OIR-resulted global changes of miRNA levels, we found that murine *Alu*-like B1/B2 elements were up-regulated in the OIR retinas associated with decreased *Dicer1* (Fig. 4e), indicating a potential role of *Dicer*-deficiency-induced *Alu* element accumulation in retinal neovascularization and degeneration in proliferative retinopathy.

Using bioinformatics tools, we analyzed putative mRNA targets of miRNAs identified in OIR retinas. Our analyses showed that these significant altered miRNAs may target multiple genes associated with several angiogenic- and hypoxic-related GO categories and angiogenic signaling pathways, including VEGF, ANGPT-TIE, and HIF pathways (Fig. 5). In addition, among the differentially expressed miRNAs, *miR-145*, *-155*, *-210*, *-375* and *-449a* are known as hypoxia-regulated miRNAs (hypoxamiRs), implicated in regulating several hypoxia-sensitive cellular responses by targeting genes involved in angiogenesis, cell cycle, and inflammation^39^,^40^,^41^. In line with our findings, up-regulation of *miR-210*, a master hypoxamiR regulated by HIF, can target ephrin-A3 (*Efna3*) to promote angiogenesis during hypoxia^39^. Similarly, increased expression of another HIF-dependent miRNA—*miR-155*— in mouse OIR model can target *CCN1*, a cysteine-rich and integrin-binding matricellular protein, to disturb the normal retinal vessel growth^35^. Consistently, our *in silico* analysis also showed that *miR-155* was up-regulated in OIR retinas and may target several genes associated with angiogenesis, inflammation and metabolism in response to hypoxia. *MiR-145* was reported to regulate differentiation and cytoskeletal dynamics of smooth muscle cells during vascular diseases^42^, and control of apoptosis in tumors^43^,^44^. In experimental models of cerebral ischemia, *miR-145* showed significant increase^45^,^46^, suggesting that hypoxia may play roles in promoting *miR-145* expression.

The present study focused on the second proliferative phase of OIR with neovascularization, with retinal samples collected at P17, the time point showing maximal pathologic neovessels^47^. Our miRNA findings at P17 were compared with a previous study in OIR at P15^48^ in order to evaluate the dynamics of differential miRNA expression (Table S3). This comparison demonstrated different groups of miRNAs preferentially altered at different time points in OIR, yet several miRNAs, including *miR-150*, *miR-375*, *miR-129-5p and miR-129-3p* showed consistent pattern of down-regulation at both P15 and P17. *MiR-150* is selectively expressed in mature B and T cells as well as abundant in vascular endothelial cells^49^,^50^. Besides, in mouse pups undergone prolong hyperoxic condition, *miR-150* showed down-regulated in the lungs^51^,^52^,^53^. Our previous study showed that endothelial *miR-150* targets angiogenic genes, such as *Fzd4* and *Dll4* as validated by 3′UTR luciferase assays, to exert an intrinsic suppressive role in pathologic retinal angiogenesis^50^. In addition, *miR-375* was observed down-regulated in the murine retinal ischemic model^35^,^48^. *Mir-375* is required for normal pancreatic development and also influences glucose-stimulated insulin secretion^54^,^55^, indicting its crucial roles in diabetes. Our data of decreased *miR-375* in OIR retinas suggest a potential additional role of *miR-375* in proliferative retinopathy.

The retina is a complex tissue consisting of several cell types, including neurons, glia, and endothelial cells. Different type of retinal cells may have specific sncRNA expression profiles. For instance, we have identified *miR-150* as an endothelial-enriched miRNA in retinal vessels and its down-regulation leads to pathologic neovascularization^50^. Moreover, a recent study characterizing the miRNome of the human retina identified retinal enrichment of three miRNAs *hsa-miR-125a-5p*, *-145-5p*, and *-486-5p*^56^, which are the human homologs of mouse *miR-351*, *-145*, and *-486* respectively, all significantly altered miRNAs in our study. Among them, *hsa-miR-125a-5p* and *-145-5p* revealed association with DR and AMD according to our analysis (Table 3), strengthening their retinal-enriched features. In addition, Arora *et al*.^56^ reported that *mmu-miR-125*, which contain identical seed sequence region as *mmu-miR-351*, was strongly expressed in the inner plexiform layer in developing mouse retina, and in the inner and outer nuclear layers in adult retinas, suggesting that *mmu-miR-351* might be expressed in retinal neurons, including photoreceptors. Investigation of Müller glia and photoreceptors in developing mouse retinas also highlighted *miR-145* as one of Müller glia-enriched miRNAs^57^.

By examining the significantly altered miRNAs of OIR retinas, we have identified 38 genes with the capacity to act as targets of the OIR-responsive miRNAs in a perturbation that leads to pathologic angiogenesis in human specimens from neovascular eye diseases (Table 3). Our findings are aligned with miRNA target enrichment in GO biologic processes of transcriptional regulation and development (Tables S1 and S2). Progression from the animal model of OIR-responsive miRNAs, to the experimentally confirmed targets of the homologous human miRNAs with differential expression profile in retinal and choroidal vascular specimens, has given us a basis to infer that human homologs of these OIR-responsive miRNAs are likely to act in pathologic retinal angiogenesis in clinical populations.

Besides miRNAs, we also observed differential expression of nineteen snoRNAs in OIR mouse retinas (Fig. 3 and Table 2). To date, most snoRNAs have no verified targets, yet some snoRNAs display up-regulation in cancers and are involved in tumorigenicity^58^. On the other hand, loss of snoRNA could also be pathogenic^14^,^15^. For instance, Prader-Willi syndrome, a severe hereditary disorder, is caused by deletion of the chromosome 15q11-q13 containing C/D box snoRNA *SNORD115* and *SNORD116*^14^. Furthermore, snoRNAs may also function as miRNA precursors, and are processed to miRNA-liked fragments to regulate gene expression^59^. In our study, while we found a globally up-regulated snoRNA expressing pattern in OIR mouse retinas, the exact role of each altered snoRNA in retinopathy still awaits further investigation.

In conclusion, our study elucidates two groups of sncRNAs—miRNAs and snoRNAs—whose expression pattern is altered in the mouse model of proliferative retinopathy. We present global miRNA reduction along with *Dicer1* decrease and *Alu* RNA increase, all of which may be potentially attributable to retinal hypoxia and ischemia in OIR. MiRNAs are key mediators of post-transcriptional regulation for fine-tuning gene expression; whereas snoRNAs are crucial in regulating protein synthesis and cell behavior, dysregulation of either or both groups of sncRNAs may exacerbate proliferative retinopathy. Our findings provide practical guiding information for deciphering the specific contribution of sncRNA processing pathway and each identified sncRNA in regulating pathological ocular angiogenesis, and potential RNA-based biomarkers and future treatments for eye diseases.

## Methods

### Animals

C57BL/6J mice were obtained from The Jackson Laboratory (stock no. 000064; Bar Harbor, ME, USA). All animal experimental procedures were approved by the Boston Children’s Hospital Institutional Animal Care and Use Committee and conducted in accordance with the Association for Research in Vision and Ophthalmology Statement for the Use of Animals in Ophthalmic and Vision Research.

### Oxygen-induced retinopathy mouse model

OIR was generated as previously described^10^ (Fig. 1a). Mouse pups and their nursing mother were exposed to 75% oxygen from postnatal day (P) 7 to P12, and returned to room air. Age-matched normoxic control mouse pups were kept in room air throughout the experiments. Mice were sacrificed at P16 or P17, followed by retinal dissection and RNA extraction.

### Small RNA isolation and miRNA microarray

Total retinal RNA including miRNAs and other small RNAs were isolated from P17 mouse OIR and normoxic retinas with miRNeasy Micro Kit (Qiagen, Chatsworth, MD, USA) according to the manufacturer’s instruction (n = 3 per group). 100 ng total RNA per sample was applied to the Affymetrix GeneChip miRNA 2.0 array (Santa Clara, CA, USA), which covers 100% miRBase v15 contents, in order to examine sncRNA expression profiles in OIR retinas and normoxic controls. Each sample is a biological replicate (n = 3 per group). The array study, from hybridization, RNA labeling, to raw data normalization, was performed by the Harvard Medical School Biopolymers Facility. For the array, the mouse miRNA probe set encompassed 722 mature miRNAs and 690 pre-miRNAs. It also contained 2,302 snoRNAs from human. The expression data were analyzed with the Affymetrix miRNA QCTool software for quality control and background analysis. Pre-miRNA, mature miRNA and snoRNA probes were identified according to annotations provided by Affymetrix. A *t*-test was applied on the mean expression values of sncRNAs for significance calculations. The resulting significant genes were grouped. Heat maps demonstrating differential sncRNA expression were generated by using the Heatmap Builder software as previously described^60^. The array data were deposited in the NCBI Gene Expression Omnibus (GEO) database (http://www.ncbi.nlm.nih.gov/geo/) for public access (accession number: GSE84303).

### Identification of putative targets of OIR-responsive miRNAs

Potential target genes of selective miRNAs were predicted according to the algorithms of TargetScan v.7 (http://www.targetscan.org)^21^ and microRNA.org (miRanada-mirSVR) (http://www.microrna.org/microrna/home.do)^22^ with incorporation of context score ++<−0.2 and mirSVR score <−0.5, respectively. Functional enrichment analysis was conducted using GeneCodis3^23^ with ontology class “Biological Process” for gene annotation. Hits of GO terms were considered according to corrected hypergeometric distributions P-values which were less than 0.05. Based on GO categories, miRNA-target gene regulatory networks were illustrated with direct interaction using Cytoscape (version 3.2.1)^61^.

### Identification of OIR-responsive miRNA targets in human neovascular eye disease transcriptome profile

For identifying validated targets of selective miRNAs in human eye diseases, we used miRTarBase 6.1 (mirtarbase.mbc.nctu.edu.tw) to obtain a set of experimentally validated human homologous miRNA targets of the mouse OIR-responsive miRNAs. TargetScan v.7 Human was used as the source for predicted miRNA-transcript interactions, applying a total context++ score value <−0.2 as a threshold for strongly predicted sites. Transcriptome analysis were performed in microarray data from human specimen with fibrovascular membranes from proliferative diabetic retinopathy patients^24^ (data obtained from GEO, GSE60436); and choroidal neovascular membranes of patients with neovascular age-related macular degeneration (AMD)^25^ (GEO:GSE29801).

### Quantitative real-time reverse transcription polymerase chain reaction (q-PCR)

Total RNAs were extracted from retinas of mice with OIR and normoxic controls at P16 using RNeasy Kit (Qiagen), and cDNAs were generated using SuperScript^®^ III Reverse Transcriptase (Invitrogen, Thermo Fisher Scientific Inc., Waltham, MA, USA) according to manufacturer’s instructions. Q-PCR was used to measure selected gene expression by using a CFX96™ Real-Time PCR Detection System (Bio-Rad, Hercules, CA, USA) and the SYBR Green Master Mix kit (Kapa Biosystems, Wilmington, MA, USA) with specific primers. Expression levels of the selected genes were normalized to 18S ribosomal RNA (*18S*) as the endogenous reference. Fold-change of expression values was calculated using the ΔΔCt method. The primer sequences used are listed below. Mouse *18S*: forward 5′-ACGGAAGGGCACCACCAGGA-3′, reverse 5’–CACCACCACCCACGGAATCG-3′; *B1*: forward 5′-TGCCTTTAATCCCAGCACTT-3′, reverse 5′-GCTGCTCACACAAGGTTGAA-3′; *B2*: forward 5′-GAGTTCAAATCCCAGCAACCA-3′, reverse 5′-AAGAGGGTCTCAGATCTTGTTACAGA-3′; *Dicer1*: forward 5′-GGTCCTTTCTTTGGACTGCCA-3′, reverse 5′-GCGATGAACGTCTTCCCTGA-3’; *Drosha*: forward 5′-AGCCGTGGAGGGTGTTATAG-3′, reverse 5′-TCCGCTCACGATGTAGGTTC-3′.

### Statistical analysis

The statistical significance of differences between mean values of two groups was computed by Microsoft Excel and assessed using Student’s *t*-test. *P* < 0.05 was considered significant.

## Additional Information

**How to cite this article**: Liu, C.-H. *et al*. Retinal expression of small non-coding RNAs in a murine model of proliferative retinopathy. *Sci. Rep.* **6**, 33947; doi: 10.1038/srep33947 (2016).

## Supplementary Material

Supplementary Information

## Figures and Tables

**Table 1:** MiRNAs with Significantly Altered Expression in OIR Retinas.

miRNA Name	miRBase Accession	Fold Change	Log_2_ (Fold Change)	*P*-value
Up-regulation				
*mmu-miR-351*	MIMAT0000609	2.388	1.256	0.0011
*mmu-miR-762*	MIMAT0003892	1.958	0.969	0.0173
*mmu-miR-210*	MIMAT0000658	1.610	0.687	0.0015
*mmu-miR-145*	MIMAT0000157	1.609	0.686	0.0016
*mmu-miR-486*	MIMAT0003130	1.598	0.676	0.0472
*mmu-miR-339*	MIMAT0000584	1.362	0.445	0.0454
*mmu-miR-34c*	MIMAT0000381	1.361	0.445	0.0407
*mmu-miR-155*	MIMAT0000165	1.295	0.373	0.0395
Down-regulation				
*mmu-miR-129-5p*	MIMAT0000209	0.511	−0.967	0.0020
*mmu-miR-150*	MIMAT0000160	0.585	−0.774	0.0171
*mmu-miR-375*	MIMAT0000739	0.642	−0.640	0.0251
*mmu-miR-203*	MIMAT0000236	0.671	−0.575	0.0403
*mmu-miR-129-3p*	MIMAT0000544	0.677	−0.563	0.0135
*mmu-miR-449a*	MIMAT0001542	0.699	−0.516	0.0462
*mmu-miR-383*	MIMAT0000748	0.707	−0.500	0.0067
*mmu-miR-1907*	MIMAT0007876	0.720	−0.474	0.0398
*mmu-miR-409*	MIMAT0001090	0.726	−0.462	0.0252

Data were filtered by fold change >1.25 or <0.75 (log_2_ ratio > 0.32 or <−0.42) and *P* < 0.05.

**Table 2:** SnoRNAs with Significantly Altered Expression in OIR Retinas.

Gene Name	Ensembl Gene ID	HGNC Symbol	Target RNA	Fold Change	*P*-value	Regulation
C/D box						
*14q (II-10)*	ENSG00000200279	SNORD114-10	unknown	1.39	0.007	Up
*HBII-52-45*	ENSG00000212380	SNORD115-45	unknown	1.28	0.015	Up
*U3*	ENSG00000221461	RNU3P2	unknown	1.28	0.022	Up
*HBII-52-8*	ENSG00000200726	SNORD115-8	Serotonin receptor 5HT-2C^62^	1.27	0.035	Up
*HBII-296B*	ENSG00000212552	SNORD91B	28S rRNA G4588^63^	1.27	0.023	Up
*U36C*	ENSG00000252542	SNORD36C	28S rRNA A3703^64^	1.27	0.026	Up
*U38A*	ENSG00000202031	SNORD38A	28S rRNA A1858^64^	1.27	0.004	Up
*U50B* (*SNHG5*)	ENSG00000203875	SNORD50B	unknown	1.26	0.032	Up
*HBII-85-9*	ENSG00000206727	SNORD116-9	unknown	1.22	0.040	Up
*U46*	ENSG00000201009	SNORD46	28S rRNA A3739^17^,^64^	0.79	0.007	Down
H/ACA box						
*U19*	ENSG00000223111	SNORA74	28S rRNA U3741^16^	1.20	0.013	Up
			28S rRNA U3743^65^			
			U3 snRNA U8^66^			
*HBI-6*	ENSG00000212624	SNORA26	28S rRNA U4522^63^	0.78	0.023	Down
Other snoRNAs						
*snoU13.418-201*	ENSG00000239159	N/A	unknown	1.28	0.020	Up
*snoU13.329-201*	ENSG00000238982	N/A	unknown	1.28	0.025	Up
*snoU13.341-201*	ENSG00000239011	N/A	unknown	1.21	0.012	Up
*snoZ13_snr52*	ENSG00000251847	N/A	unknown	1.21	0.045	Up
*snosnR60_Z15*	ENSG00000201853	N/A	unknown	0.80	0.026	Down
*snoZ5*	ENSG00000251721	N/A	unknown	0.79	0.039	Down
*snoU13.25-201*	ENSG00000238336	N/A	unknown	0.76	0.040	Down

Data were filtered by fold change >1.20 or <0.75 and *P* < 0.05.

**Table 3:** OIR-responsive miRNAs and Disease-Associated Altered Expression of Genes Encoding Pertinent Experimentally Verified Targets.

miRNA ID		Verified Human Targets*	miRNA Target Genes with Altered Expression in Diseased Human Retinal Tissue^24^,^25^			
						PDR	NV AMD *vs.* No AMD	
Mouse	Human		PDR *vs.* No PDR	Active *vs.* Inactive FVM	RPE/Choroid	Neural Retina		
Up-regulation in OIR						
*mmu-miR-351-5p*	*hsa-miR-125a-5p*#	32	*CDK1NA*, *CLEK5A*	*CD34*	N/A	*ARID3B*, *ERRB2*
*mmu-miR-762*	*hsa-miR-762*	2	N/A	N/A	N/A	N/A
*mmu-miR-210-3p*	*hsa-miR-210-3p*	60	*COL4A2*	N/A	*MNT*	N/A
*mmu-miR-145a-5p*	*hsa-miR-145-5p*	164	*CDH2*, *CDK6*, *CDKN1A*, *COL5A1*, *SERPINE1*, *NDRG2*	*ANGPT2*, *ETS1*, *SOX2*	*CTGF*, *ITGB8*, *MEST1*, *MMP1*	*CLINT1*, *MYO5A*
*mmu-miR-486a-5p*	*hsa-miR-486-5p*	4	N/A	N/A	N/A	N/A
*mmu-miR-339-5p*	*hsa-miR-339-5p*	3	N/A	N/A	N/A	N/A
*mmu-miR-34c-5p*	*hsa-miR-34c-5p*	44	N/A	*SOX2*	N/A	N/A
*mmu-miR-155-5p*	*hsa-miR-155-5p*	294	N/A	*DOCK1*, *ETS1*, *IL17RB*	*NOVA1*	*CKAP5*, *FADD*, *FAM177A1*, *MYO10*
Down-regulation in OIR						
*mmu-miR-129-5p*	*hsa-miR-129-5p*	15	*CDK6*	N/A	*ABCC5*	*SOX4*
*mmu-miR-150-5p*	*hsa-miR-150-5p*	34	N/A	*NOTCH3*, *IGF2*	*IGF1*	N/A
*mmu-miR-375-3p*	*hsa-miR-375*	39	N/A	*RASD1*	N/A	*ERRB2*
*mmu-miR-203-3p*	*hsa-miR-203a-3p*	63	*CDK6*	N/A	N/A	N/A
*mmu-miR-129-2-3p*	*hsa-miR-129-2-3p*	4	*CDK6*	N/A	N/A	*SOX4*
*mmu-miR-449a-5p*	*hsa-miR-449a*	30	*CDK6*, *LEF1*	N/A	N/A	N/A
*mmu-miR-383-5p*	*hsa-miR-383-5p*	8	*IRF1*	N/A	N/A	N/A
*mmu-miR-1907*	N/A	N/A	N/A	N/A	N/A	N/A
*mmu-miR-409-3p*	*hsa-miR-409-3p*	8	N/A	N/A	*FGG*	N/A

AMD, age-related macular degeneration; FVM, fibrovascular membrane; N/A, not applicable; NV, neovascular; PDR, proliferative diabetic retinopathy.

^*^Experimentally validated miRNA human target interactions from qPCR, luciferase reporter assays, and Western blots were obtained from mirTarBase 6.1.

^#^*Hsa-miR-125a-5p* contains identical seed sequence region as *mmu-miR-351-5p*, hence it was analyzed as the human homolog.
