# Supplementary material for: Retinal expression of small non-coding RNAs in a murine model of proliferative retinopathy
Source: Sci Rep. 2016 Sep 22;6:33947. doi: 10.1038/srep33947 (PMC5032015; doi:10.1038/srep33947)
Supplement: Supplementary Information [file srep33947-s1.pdf]

Supporting Information

Retinal expression of small non-coding RNAs in a murine model of proliferative retinopathy

Chi-Hsiu Liu[1], Zhongxiao Wang[1], Ye Sun[1], John Paul SanGiovanni[2], Jing Chen[1, *]

[1]Department of Ophthalmology, Boston Children's Hospital, Harvard Medical School, Boston, Massachusetts 02115, USA; [2]Section on Nutritional Neuroscience, National Institute on Alcohol Abuse and Alcoholism, Bethesda, MD 20892, USA,

*Corresponding author: Jing Chen, email: jing.chen@childrens.harvard.edu

Supplementary information — Additional detailed methods:

Identification of Genes Differentially Expressed in Human Retinal Specimens with Pathologic Angiogenesis of the Retinal and Choroidal Vasculature

We used extant comparative transcriptomic profiles from retinal specimens containing: 1) active and quiescent fibrovascular membranes (FVMs) donated by people with proliferative diabetic retinopathy (PDR)[1]; and, 2) choroidal neovascular (CNV) membranes donated by people with neovascular age-related macular degeneration (NV AMD)[2] to search for overlap with constructs containing strong experimental evidence of interaction with the 17 OIR-responsive miRNAs. FVM-PDR and CNV-NV AMD candidate molecules were derived from comparison of findings from age-matched disease-free retinas. Microarray expression data were downloaded from Gene Expression Omnibus (GEO), GSE60436 and GSE29801 for PDR and AMD samples respectively.

FVM-PDR Specimens.[1] The PDR-related retinal transcriptome was identified through multi-dimensional false-discovery control procedures (FDR2D) on normalized microarray data that showed differential expression in diseased tissue at *Q*-values ≤ 0.15. Analysis included specimens from 9 eyes (3 active FVMs, 3 inactive FVMs, and 3 healthy retinas). FVMs were classified as active if perfused preretinal capillaries existed. Inactive FVMs were defined as those with: 1) previously documented active proliferation that had regressed fully; or 2) only nonperfused, gliotic, or fibrotic vessels. The human WG-6 V3 Expression BeadChips (Illumina, San Diego, CA, USA) microarray platform was run on the 250 ng of isolated total RNA was converted to biotinylated-cRNA. Normalized values were calculated with GeneSpring GX 11.0 software (Agilent Technologies, Santa Clara, CA, USA).

There were 87 and 89 respective genes expressed at significantly higher and lower levels in FVMs (vs. disease-free specimens). There were 91 and 89 respective genes with over and under expression within active (vs. inactive) FVMs[1].

CNV-NV AMD Specimens.[2] The NV AMD-related transcriptome was identified through permutation testing with the Fisher-Yates method – genes that showed FDR values $\leq$ 0.02, permuted *P* values $\leq$ 0.1, and fold-change $\geq$ 1.5 were identified as candidates for classification into NV AMD-related modules (gene groups that exhibited coordinated expression in NV AMD and functional enrichment in one or more biological processes). Analyses were conducted on normalized values reported from specimens of RPE-choroid and retina from 31 normal and 4 NV AMD human donor eyes with the Agilent Whole Human Genome 4 × 44 K *in situ* oligonucleotide array platform (G4112F, Agilent Technologies, Inc., Santa Clara, CA, USA). The NV AMD phenotype was defined as AREDS Level 4 and Rotterdam Grade 4. The comparison group had not patent cardinal features of AMD.

There were 391 and 310 respective genes of NV AMD-associated coordinated expression modules expressed at significantly higher and lower levels in the neural retina of people with CNV (vs. disease-free specimens). There were 206 and 96 respective genes with over and under expression within the RPE/choroid of people with CNV (vs. disease-free specimens) [2].

Functional Annotation were performed with GeneCodis3[3] and the PANTHER Overrepresentation Test (release 20160321, http://geneontology.org/) for additional functional annotations from the Gene Ontology Consortium resource center.

Supplementary information — References:

1 Ishikawa, K. *et al.* Microarray analysis of gene expression in fibrovascular membranes excised from patients with proliferative diabetic retinopathy. *Investigative ophthalmology & visual science* **56**, 932-946, doi:10.1167/iovs.14-15589 (2015).

2 Newman, A. M. *et al.* Systems-level analysis of age-related macular degeneration reveals global biomarkers and phenotype-specific functional networks. *Genome medicine* **4**, 16, doi:10.1186/gm315 (2012).

3 Tabas-Madrid, D., Nogales-Cadenas, R. & Pascual-Montano, A. GeneCodis3: a non-redundant and modular enrichment analysis tool for functional genomics. *Nucleic acids research* **40**, W478-483, doi:10.1093/nar/gks402 (2012).

4 Shen, J. *et al.* MicroRNAs regulate ocular neovascularization. *Molecular therapy : the journal of the American Society of Gene Therapy* **16**, 1208-1216, doi:10.1038/mt.2008.104 (2008).

Number of Supplemental Tables: 3

Supplementary information — Tables

Table S1. Enrichment Analysis of Pertinent Experimentally Verified Targets of OIR-responsive miRNAs in Biological Process

GO ID	Term	*P* value	Genes
GO:0007596	Blood coagulation	0.000127	*FGG,ANGPT2,IRF1,MMP1,DOCK1,IGF1,SERPINE1*
GO:0045944	Positive regulation of transcription from RNA polymerase II promoter	0.000152	*LEF1,SOX4,IGF2,SOX2,IRF1,IGF1,ETS1*
GO:0007275	Multicellular organismal development	0.001358	*IGF2,SOX2,ITGB8,ANGPT2,NOTCH3,NDRG2,MNT*
GO:0007165	Signal transduction	0.002269	*MYO10,FGG,ANGPT2,NDRG2,RASD1,DOCK1,IGF1*
GO:0008285	Negative regulation of cell proliferation	0.000148	*SOX4,CDKN1A,MNT,IGF1,CDK6,ETS1*
GO:0008284	Positive regulation of cell proliferation	0.000123	*LEF1,SOX4,IGF2,IGF1,ETS1,CTGF*
GO:0007155	Cell adhesion	0.000809	*LEF1,CDH2,ITGB8,CD34,CTGF,COL5A1*
GO:0006355	Regulation of transcription, DNA-dependent	0.013481	*SOX4,ARID3B,IGF2,SOX2,NOTCH3,MNT*
GO:0045893	Positive regulation of transcription, DNA-dependent	0.001817	*LEF1,SOX4,SOX2,IGF1,ETS1*
GO:0043410	Positive regulation of MAPK cascade	9.05E-05	*IGF2,CDH2,SOX2,IGF1*
GO:0014070	Response to organic cyclic compound	0.000346	*LEF1,CDKN1A,ANGPT2,CTGF*
GO:0043066	Negative regulation of apoptotic process	0.002383	*LEF1,SOX4,CDKN1A,SERPINE1*
GO:0007411	Axon guidance	0.003115	*MYO10,COL4A2,DOCK1,COL5A1*
GO:0048146	Positive regulation of fibroblast proliferation	0.000507	*CDKN1A,IGF1,CDK6*
GO:0001649	Osteoblast differentiation	0.000598	*LEF1,IGF2,SOX2*
GO:0031100	Organ regeneration	0.000902	*LEF1,CDKN1A,ANGPT2*
GO:0090090	Negative regulation of canonical WNT receptor signaling pathway	0.001265	*LEF1,CDH2,SOX2*
GO:0045766	Positive regulation of angiogenesis	0.001112	*ANGPT2,SERPINE1,ETS1*
GO:0009611	Response to wounding	0.001112	*SOX2,ETS1,CTGF*
GO:0002576	Platelet degranulation	0.001574	*FGG,IGF1,SERPINE1*
GO:0032355	Response to estradiol stimulus	0.001778	*IGF2,ETS1,CTGF*
GO:0016477	Cell migration	0.002402	*CDH2,DOCK1,COL5A1*
GO:0050900	Leukocyte migration	0.002388	*ANGPT2,CD34,MMP1*
GO:0007050	Cell cycle arrest	0.003454	*CDKN1A,SOX2,CDK6*
GO:0001501	Skeletal system development	0.003663	*SOX4,IGF2,IGF1*
GO:0030168	Platelet activation	0.009971	*FGG,IGF1,SERPINE1*
GO:0006366	Transcription from RNA polymerase II promoter	0.010967	*IRF1,MNT,ETS1*
GO:0000278	Mitotic cell cycle	0.011857	*CDKN1A,CKAP5,CDK6*
GO:0031017	Exocrine pancreas development	0.001174	*IGF2,IGF1*
GO:0021542	Dentate gyrus development	0.001191	*LEF1,CDK6*

Table S2. Enrichment Analysis of Pertinent Experimentally Verified Targets of OIR-responsive miRNAs in Kyoto Encyclopedia of Genes and Genomes (KEGG) database

KEGG ID	Pathway	*P* value	Genes
Kegg:05200	Pathways in cancer	4.79E-08	*CDK6,LEF1,IGF1,COL4A2,ETS1,FADD,CDKN1A,MMP1*
Kegg:04510	Focal adhesion	2.68E-05	*DOCK1,IGF1,COL4A2,COL5A1,ITGB8*
Kegg:04115	p53 signaling pathway	1.64E-05	*CDK6,IGF1,SERPINE1,CDKN1A*
Kegg:04514	Cell adhesion molecules	0.001443	*CD34,CDH2,ITGB8*
Kegg:05412	Arrhythmogenic right ventricular cardiomyopathy	0.000488	*LEF1,CDH2,ITGB8*
Kegg:04512	ECM-receptor interaction	0.000636	*COL4A2,COL5A1,ITGB8*
Kegg:05215	Prostate cancer	0.000639	*LEF1,IGF1,CDKN1A*
Kegg:05218	Melanoma	0.000516	*CDK6,IGF1,CDKN1A*
Kegg:05214	Glioma	0.00047	*CDK6,IGF1,CDKN1A*
Kegg:04610	Complement and coagulation cascades	0.008789	*FGG,SERPINE1*
Kegg:05160	Hepatitis C	0.02032	*CDKN1A,IRF1*
Kegg:05410	Hypertrophic cardiomyopathy	0.011109	*IGF1,ITGB8*
Kegg:04974	Protein digestion and absorption	0.010789	*COL4A2,COL5A1*
Kegg:04810	Regulation of actin cytoskeleton	0.045826	*DOCK1,ITGB8*
Kegg:04320	Dorso-ventral axis formation	0.001463	*ETS1,NOTCH3*
Kegg:05414	Dilated cardiomyopathy	0.011508	*IGF1,ITGB8*
Kegg:05219	Bladder cancer	0.004026	*CDKN1A,MMP1*
Kegg:04110	Cell cycle	0.01835	*CDK6,CDKN1A*
Kegg:05222	Small cell lung cancer	0.010918	*CDK6,COL4A2*
Kegg:05142	Chagas disease (American trypanosomiasis)	0.013435	*SERPINE1,FADD*
Kegg:05220	Chronic myeloid leukemia	0.0102	*CDK6,CDKN1A*
Kegg:05146	Amoebiasis	0.013435	*COL4A2,COL5A1*

Table S3. Expression of Selected Retinal miRNAs at Different Time Points in the Mouse OIR model

Time Point	P15 (Shen et al.[4])	P17
**Mouse Strain**	C57BL/6	C57BL/6J
**Platform/Supplier of Array**	LC Sciences Microarray Service	Affymetrix GeneChip miRNA 2.0 array
**Up-regulated miRNA**	*miR-451*	*miR-351*
	miR-214	*miR-762*
	miR-424	*miR-210*
	miR-199a	*miR-145*
	miR-146	*miR-486*
	miR-106a	*miR-339*
	miR-350	*miR-34c*
	miR-210	*miR-155*
**Down-regulation miRNA**	*miR-184*	*miR-129-5p*
	miR-31	*miR-150*
	miR-150	*miR-375*
	miR-409	*miR-203*
	miR-375	*miR-129-3p*
	miR-129-5p	*miR-449a*
	miR-124a	*miR-383*
	miR-29a	*miR-1907*
	miR-129-3p	*miR-409*
